# Histology-informed multiscale modeling of human brain white matter

**DOI:** 10.1038/s41598-023-46600-3

**Published:** 2023-11-10

**Authors:** Saeideh Saeidi, Manuel P. Kainz, Misael Dalbosco, Michele Terzano, Gerhard A. Holzapfel

**Affiliations:** 1 Institute of Biomechanics, Graz University of Technology, Graz, Austria; 2 GRANTE - Department of Mechanical Engineering, Federal University of Santa Catarina, Florianópolis, SC Brazil; 3 Department of Structural Engineering, Norwegian University of Science and Technology (NTNU), Trondheim, Norway

**Keywords:** Engineering, Biomedical engineering

## Abstract

In this study, we propose a novel micromechanical model for the brain white matter, which is described as a heterogeneous material with a complex network of axon fibers embedded in a soft ground matrix. We developed this model in the framework of RVE-based multiscale theories in combination with the finite element method and the embedded element technique for embedding the fibers. Microstructural features such as axon diameter, orientation and tortuosity are incorporated into the model through distributions derived from histological data. The constitutive law of both the fibers and the matrix is described by isotropic one-term Ogden functions. The hyperelastic response of the tissue is derived by homogenizing the microscopic stress fields with multiscale boundary conditions to ensure kinematic compatibility. The macroscale homogenized stress is employed in an inverse parameter identification procedure to determine the hyperelastic constants of axons and ground matrix, based on experiments on human corpus callosum. Our results demonstrate the fundamental effect of axon tortuosity on the mechanical behavior of the brain’s white matter. By combining histological information with the multiscale theory, the proposed framework can substantially contribute to the understanding of mechanotransduction phenomena, shed light on the biomechanics of a healthy brain, and potentially provide insights into neurodegenerative processes.

## Introduction

In the human brain, white and gray matter form a highly inhomogeneous structure, which leads to region-dependent mechanical behavior^1, 2^. Gray matter consists primarily of neuronal cell bodies, whereas white matter is characterized by a more complex microstructure, including oligodendrocytes, fibrous astrocytes, microglia, capillaries, and a dense network of myelinated axon fibers embedded in a soft extracellular matrix^3^. There is evidence that pathological and physiological changes in the brain microstructure, such as those resulting from traumatic brain injury, growing tumors, neurodegenerative disorders including Alzheimer’s disease, or even healthy aging, directly affect the mechanical properties of brain tissue^4–9^. In addition, recent studies have shown that growth and development of axons are not only related to chemical but also to mechanical stimuli and can be affected by changes in tissue stiffness^10–12^. Therefore, there is a need to improve our understanding of the interactions between the brain microstructure and its mechanical behavior through the development of new computational models. In this way, we could better investigate mechanotransduction phenomena that contribute to normal brain function and shed light on the underlying mechanisms of brain neurodegeneration.

Microstructure-informed computational models are developed as powerful tools that connect the microstructure of the tissue with its macroscopic mechanical response. These models can be roughly divided into two groups: (i) structure-based continuum models, in which the heterogeneous microstructure occurs indirectly in the formulation of the constitutive law^13–15^; (ii) micromechanical multiscale models, where the microstructure occurs explicitly at the element level and the macroscopic response is obtained through the homogenization of the microscopic fields. With regard to the latter, multiscale models based on representative volume elements (RVEs) were combined with numerical homogenization for fiber-reinforced soft tissues such as tendons^16^, arteries^17–19^, myocardium^20^ and brain tissue. These multiscale models adopt a physical representation of the microstructure to simulate the homogenized response of the material at the macroscale^21,22^. Multiscale models of brain tissue have been developed mainly for the white matter, with particular emphasis on the brain stem and the corpus callosum (CC). Both regions are characterized by bundles of highly oriented axon fibers^23–33^. For the brain RVE, most studies proposed a micrometer-scale cubic geometry comprising a limited number of axons modeled as straight or wavy cylindrical inclusions uniformly distributed within a ground matrix^24,27,30,33^. Diffusion tensor imaging^34^ and, more recently, three-dimensional (3D) imaging with polarized light^35^ have enabled the reconstruction of the orientation of axon fibers. A more realistic fiber architecture, including random location and orientation of the axons, was included in the model presented by Hoursan et al. ^31^.

Multiscale models provide a convenient tool to characterize the viscoelastic and hyperelastic mechanical properties of brain tissue microstructure. The macroscale response derived from the stress homogenization can be fitted to experimental data to determine the mechanical parameters of the constituents by an inverse procedure^32,33,36^. Given the difficulty of directly quantifying the material properties of axons, this is a valuable consideration. From a mechanical point of view, brain tissue exhibits a soft, time-dependent material behavior^37–40^. Neuroimaging techniques highlight a distinct structural anisotropy^34,41,42^, although there is disagreement whether this is also reflected in the macroscopic mechanical anisotropy^3,43,44^. In addition, current predictions of the hyperelastic and viscoelastic material properties of white matter are inconsistent. According to reports, axons are three to thirteen times stiffer than the ground matrix^30,31,45,46^. Histological images also indicate that the axons follow wavy streams^45,47–49^, an aspect that can have relevant implications for the performance of the micromechanical model^24,26,27^. Overall, a micromechanical model based on data obtained from histological information combined with parameter identification performed at multiple loading modes simultaneously could lead to a better understanding of the relationship between microstructure and macroscopic mechanical behavior.

The main objective of this study is to propose a new microscale RVE of the brain white matter. The micromechanical model includes several microstructural parameters, such as axon diameter, orientation and tortuosity, based on histological information. For each of these parameters, appropriate probability density functions (PDFs) are calibrated to the experimental distribution observed in human CC. Furthermore, we propose an efficient implementation in a finite element framework based on the embedded element technique. This technique, which was applied to the modeling of axon fibers^50^ and more recently to collagen networks by Dalbosco et al.^19^, is optimal for simulating complex architectures of one-dimensional (1D) fibers. The fiber tortuosity is indirectly included in the constitutive law via the concept of the recruitment stretch^13,51^. The second objective is to investigate how the microstructure correlates with the macroscopic, possibly anisotropic, mechanical response of the white matter. For this purpose, the homogenized response is used to determine the mechanical parameters of axon fibers and the ground matrix, through an inverse parameter identification scheme based on existing experiments^2^. The model is then validated against load cases other then those used to identify the material parameters. Finally, the influence of fiber tortuosity and orientation on the homogenized response of the RVE is assessed.

## Results

### Representative volume element of brain white matter

**Figure 1:**
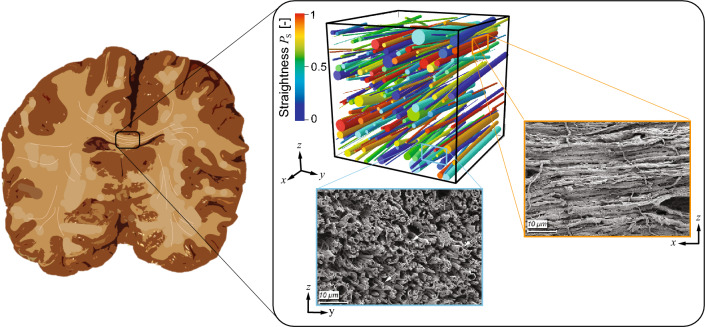
Representative volume element of the corpus callosum. View of the human brain in the coronary plane and axon fiber network in the corpus callosum from scanning electron microscopy^47^. The 3D RVE with random axon fiber network represents axons as cylindrical structures (color is related to their tortuosity).

We designed a microscopic RVE based on a simplified two-component representation of the brain white matter. A continuous 3D medium describes the ground matrix, including the extracellular matrix and the non-neuronal cells, reinforced by 1D myelinated axon fibers. Following the work from our group on arterial tissue^19^, axons are distributed in the matrix at random locations with different diameter, orientation, and tortuosity. We considered the outer diameter of axons, including the myelin sheath. Fiber tortuosity refers to the waviness of the axon fibers in the unloaded state, here implemented through the concept of recruitment stretch (see Methods). The volume of axon fibers is defined by the fiber volume fraction ^2^. An illustration of the RVE is shown in Fig. 1. The algorithm for generating the random fiber network is described in detail in the Supplementary Material available online.

We included microstructural features of axon fibers in the RVE from existing histological information of brain CC, in the form of distribution functions. Namely, we considered three parameters: fiber diameter, fiber tortuosity and fiber orientation.

**Figure 2:**
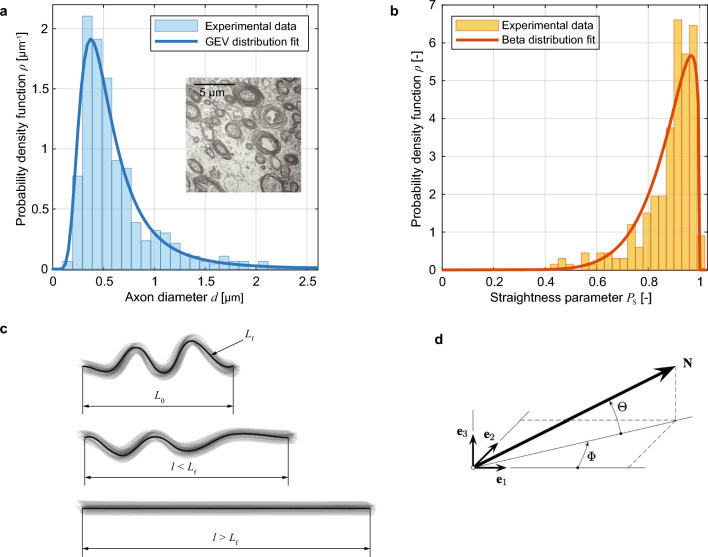
Illustration of the structural parameters considered in the model. (a) Histogram of axon diameter distribution for the human corpus callosum and the PDF fit of the generalized extreme value (GEV) based on (1) (the insert is adapted from^52^); (b) histogram of axon fiber straightness parameter $P_{\textrm s}$ for ovine corpus callosum (data adapted from^48^) and the beta distribution fit based on (2); (c) concept of fiber recruitment, with the initial tortuous configuration, the partially unbent state (fiber stretch $\lambda < 1/P_{\textrm s}$) and the recruited state ($\lambda > 1/P_{\textrm s}$); (d) orientation of a single axon fiber in space (adapted from^54^).

Scanning electron micrographs show that the diameter of myelinated axons in the human CC follows a certain distribution^52^. We fitted the experimental data of the genu (the bending of the anterior CC) to a generalized extreme value distribution defined by the following PDF

1 $$\begin{aligned} \rho ({d}|\,\mu , \sigma , \xi )=\frac{1}{\sigma }\left[ 1+\xi \left( \frac{{d}-\mu }{\sigma }\right) \right] ^{-(\frac{1}{\xi }+1)} \exp \left[ -\left( 1+\xi \frac{{d}-\mu }{\sigma }\right) ^{-\frac{1}{\xi }}\right] \,, \end{aligned}$$

where *d*, $\mu$, $\sigma$ and $\xi$ represent the fiber diameter, the location parameter, the scale parameter, and the shape parameter of the probability density, respectively^53^. The experimental distribution and fitting are shown in Fig. 2a.

From histological images of the white matter, we could observe wave-like streams, suggesting that axons can be characterized by a certain tortuosity^45,55^. In the absence of information from human brain, we employed data derived from scanning electron micrographs of sheep CC, which suggested a log-normal distribution of the axon tortuosity $\tau$ ^48^. More precisely, we defined a straightness parameter $P_{\textrm s} = 1 -\tau = {L_0}/{L_{\textrm f}}$, where $L_0$ is the end-to-end length of the fiber and $L_{\textrm f}$ denotes the arc length of the center line, measured in the reference configuration. We then fitted the experimental distribution of the straightness parameter $P_{\textrm s}$ using a beta PDF, i.e.

2 $$\begin{aligned} \rho ({P}_{\textrm s}|\,{a}, {b}) = \frac{\Gamma ({a}+{b}){P}_{\textrm s}^{{a}-1}(1-{P}_{\textrm s})^{{b}-1}}{\Gamma ({a})\Gamma ({b})}\,, \end{aligned}$$

where $\Gamma (\bullet )$ is the gamma function of $(\bullet )$ and $a,b>0$ are shape parameters. The histogram and the corresponding fit are shown in Fig. 2b. The parameter $P_{\textrm s}\rightarrow 0$ is characteristic of large fiber tortuosity, whereas $P_{\textrm s}\rightarrow 1$ refers to straighter fibers. As specified in the Methods, we modeled the axons as 1D straight segments and indirectly incorporated tortuosity into the constitutive law, based on the concept of recruitment stretch^19,51^. In summary, the stretch of a tortuous fiber consists of a first stage of unbending or fiber recruitment, with no mechanical contribution, and a subsequent phase in which the fully stretched fiber starts to bear loads. This concept is illustrated in Fig. 2c.

Finally, the orientation distribution of axon fibers in space is described by a density function, with the orientation of a single fiber identified by azimuthal and elevation angles (Fig. 2d). Following the collagen fiber dispersion model proposed by Holzapfel et al.^54^, we assumed a bivariate von Mises PDF, which requires a pair of concentration parameters $\alpha ,\beta \ge 0$ (Eq. 4 in Methods). Despite recent advances in neuroimaging methods, high-resolution images of the fibrous network in human white matter are still scarce^35,56^. In vivo diffusion tensor imaging studies can provide the orientation of the fibers with millimeter-scale resolution^34,57^, which is not applicable to the microscopic range of this work. Furthermore, increasing evidence confirms that axons in the human CC are highly unidirectional^47^. Therefore, we assumed perfect alignment of the axons ($\alpha \rightarrow \infty$, $\beta \rightarrow \infty$) for model fitting and validation. A summary of the complete structural parameters used in this work is listed in Table 1.

**Table 1:** Structural parameters of corpus callosum. The parameters of different PDFs are fitted to existing experimental data.

Parameter	Description	Value
$v_{\textrm f}$	Axon volume fraction	0.400 [–]
**Axon diameter (Generalized extreme value PDF)**		
$\mu$	Location parameter	0.426 [$\mu$m]
$\sigma$	Scale parameter	0.200 [$\mu$m]
$\xi$	Shape parameter	-0.305 [–]
**Axon tortuosity (Beta PDF)**		
*a*	Shape parameter	9.155 [–]
*b*	Shape parameter	1.275 [–]
**Axon orientation (Bivariate von Mises PDF)**		
$\alpha$	Concentration parameter	$\infty$
$\beta$	Concentration parameter	$\infty$

### Size of the representative volume element

Due to the random structure of the axon fiber network, different RVEs are generated for the unique set of characteristic PDF parameters listed in Table 1. Therefore, a fundamental step is to determine the size of the RVE, which offers a significant macroscale representation of the mechanical behavior, independently of the random network.

**Figure 3:**
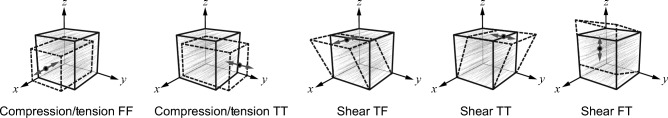
Loading modes applied to the RVE. Compression and tension parallel (FF) and transverse (TT) to the fibers, and three modes of simple shear (axon fibers are aligned with the *x*-axis).

We generated $n=10$ RVEs in four groups based on different edge lengths, $L_{\textrm a}= 5, 15, 25$ and $50\,\mu$m (40 RVEs in total), using the same structural parameter set. All RVEs are subject to the same loading, which was defined based on seven simple deformation modes used in the experiments of Budday et al ^2^. These include uniaxial compression and tension up to a stretch of 1.2 parallel and transverse the fiber direction, as well as three modes of simple shear up to an amount of shear of $\gamma = 0.2$, as illustrated in Fig. 3.

To ensure kinematic compatibility between the micro and macroscales, periodic boundary conditions are applied to the RVE ^19^ (see  Methods). The macroscale response is provided by the homogenized stress, which is calculated based on the rule of mixture and the assumption of continuity in the displacement fields of matrix and fibers (no-slip condition)^19^. The homogenized first Piola–Kirchhoff (nominal) stress ${\textbf{P}}$ is then defined as $\textbf{P} = \overline{\textbf{P}}_{\textrm m} + v_{\textrm f}{\overline{\tilde{\textbf{P}}}}_{\textrm f}$, where $v_{\textrm f}$ is the fiber volume fraction, $\overline{\textbf{P}}_{\textrm m}$ is the volume-averaged stress in the ground matrix and ${\overline{\tilde{\textbf{P}}}}_{\textrm f}$ represents the volume-averaged stress for the embedded fibers. These are obtained from

3 $$\begin{aligned} \overline{\textbf{P}}_{{\textrm m}}=\dfrac{1}{{V}_{\textrm m}}\int _{{V}_{\textrm m}}\textbf{P}_{\textrm m}{\textrm d}V, \qquad {\overline{\tilde{\textbf{P}}}}_{{\textrm f}}=\dfrac{1}{{V}_{\textrm f}}\int _{V_{\textrm f}}\tilde{\textbf{P}}_{\textrm f}{\textrm d}V, \end{aligned}$$

where ${V}_{\textrm m}$ is the volume of the ground matrix and ${V_{\textrm f}}=\sum _{i=1}^{N_{\textrm f}}{V_{\textrm f}^{(i)}}$ is the total volume of the fibers.

**Figure 4:**
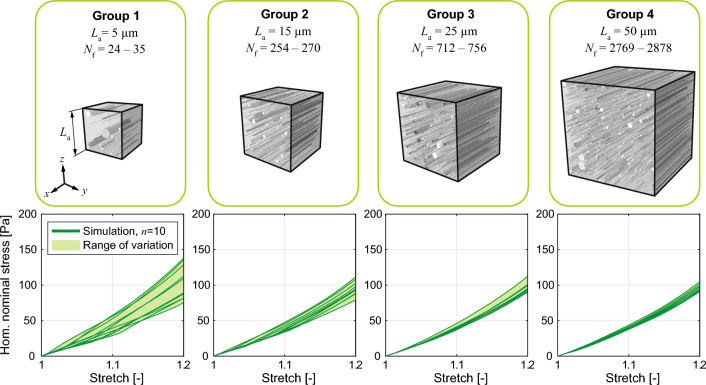
RVE size convergence. Top: groups of RVEs with four different edge lengths $L_{\textrm a}$ (${N}_{\textrm f}$ is the variation of fiber number for $n=10$ RVEs). Bottom: corresponding homogenized nominal stress plotted against the stretch from the simulation of $n = 10$ RVEs in each group (tension FF). The legend displayed in the first chart is the same for the remaining charts.

Results of the RVE size convergence analyses are illustrated in Fig. 4 in terms of homogenized stress-stretch curves from the tension FF simulations. The shaded plots show the variability range for the macroscopic response of the randomly generated RVEs. Due to the concept of recruitment stretch, perfectly aligned fibers do not provide any mechanical contribution when compressed along their axis or stretched in the perpendicular direction. Therefore, compression FF and tension TT were not considered in the convergence analysis. The size of the RVE and the fiber network have a significant impact; however, we can see a convergent pattern, suggesting that larger RVEs can provide a more accurate representation of the microstructure. Considering the high computational costs for the RVEs in group 4 and minimum differences to the results of group 3, an RVE edge size of $L_{\textrm a} = 25\,\mu$m is chosen as the optimal RVE. It is important to highlight that the homogenized stress in each group varies independently of the number of fibers in the RVE.

### Material parameter identification and model validation

The optimal RVE is used to simulate the homogeneous macroscale deformations in order to identify the material parameters of the ground matrix and of the axon fibers. We defined an inverse parameter identification algorithm (see Methods) and employed the experimental data from Budday et al.^2^ for the human brain CC, from compression FF and tension FF, TT (Fig. 3).

We assumed that the ground matrix behaves like a nearly incompressible isotropic material, whose strain-energy function $\Psi _{\textrm m}$ is given by a one-term Ogden material model, as suggested by numerous studies on the time-independent behavior of human brain under various loading conditions^2,4^. We postulated an analogous material model for the axon fibers, with a modified formulation of the strain-energy function of the single fiber $\tilde{\Psi }_{\textrm f}^{(i)}$ to include the concept of recruitment stretch and the assumption of incompressibility.

**Figure 5:**
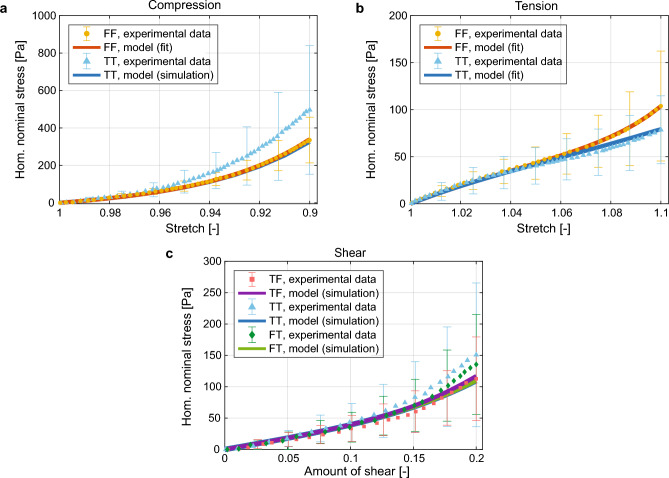
Macroscale response of the optimal RVE. Homogenized nominal stress as a function of the stretch in (a) compression and (b) tension, and as a function of the amount of shear for three simple-shear modes. Curves labeled as simulation are reproduced in the model validation phase, using the material parameters identified from the cases denoted as fit. Experimental curves adapted from^2^.

In total, four material parameters must be determined: shear moduli of ground matrix and fibers, $\mu _{\textrm m}$ and $\mu _{\textrm f}$, and two nonlinearity parameters $\alpha _{\textrm m}, \alpha _{\textrm f}$. In our modeling approach, we embedded the axon fibers inside the volume of the ground matrix, assuming perfect adhesion. Accordingly, $\tilde{\Psi }_{\textrm f}^{(i)}$ denotes a modified strain-energy function to account for a stiffness redundancy resulting from the embedding ^50^. Therefore, the material parameters $\mu _{\textrm f}$ and $\alpha _{\textrm f}$ are to be understood as parameters of the embedded fibers. More details are provided in the Methods. Figure 5 shows the model fit (labeled as ‘fit’ results) compared to the experimental stress-stretch curve in compression FF (Fig. 5a) and tension FF, TT (Fig. 5b). The material parameters identified are summarized in Table 2. In addition, the model is validated by testing its prediction capabilities in the remaining loading cases not used to identify the material parameters, namely compression TT and shear TF, FT, TT. The results are shown in Fig. 5 (labeled as ‘simulation’ results).

**Table 2:** Optimal material parameters of the ground matrix and the embedded axon fibers.

Ground matrix		Embedded axon fibers	
$\mu _{\textrm m}$ [Pa]	$\alpha _{\textrm m}$ [–]	$\mu _{\textrm f}$ [Pa]	$\alpha _{\textrm f}$ [–]
353.5	$-21.5$	80.8	62.3

### Effect of fiber orientation

**Figure 6:**
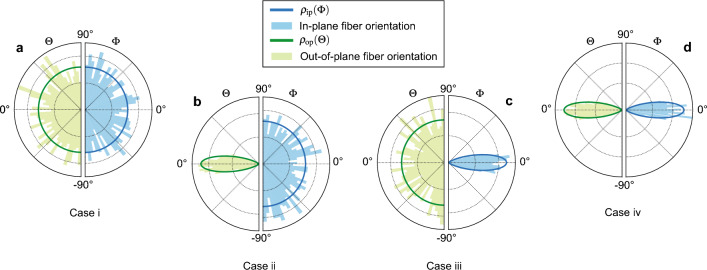
Visualization of fiber orientation. Polar plots of in-plane and out-of-plane fiber distributions in the RVE (histograms), with solid curves showing the corresponding PDF: (a) Case i: in-plane and out-of-plane isotropy; (b) case ii: planar isotropy and out-of-plane orientation; (c) case iii: in-plane orientation and out-of-plane isotropy; (d) case iv: in-plane and out-of-plane orientation. The spherical angles $\Phi ,\Theta$ are illustrated in Fig. 2d.

**Figure 7:**
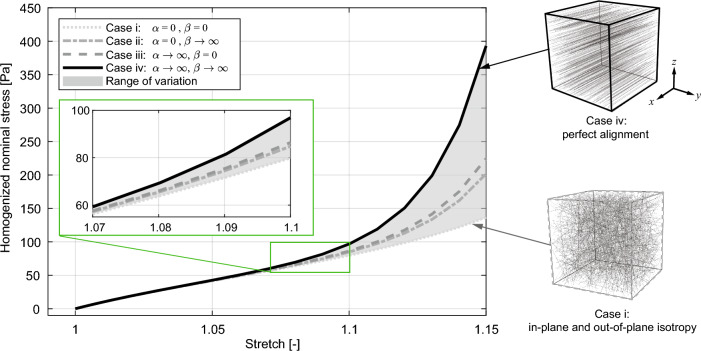
Effect of axon fiber orientation. Homogenized nominal stress versus stretch for tension FF (parallel to the global *x*-axis), considering four fiber dispersion cases listed in Table 3.

We performed analyses on RVEs with edge length $L_{\textrm a} = 25\,\mu$m using the optimized material parameters listed in Table 2 but changing the concentration parameters $\alpha$ (in-plane) and $\beta$ (out-of-plane) to assess the influence of the fiber orientation. Four different cases, summarized in Table 3 and illustrated in Fig. 6, were considered: (i) in-plane and out-of-plane isotropy; (ii) plane isotropy and out-of-plane alignment; (iii) in-plane alignment and out-of-plane isotropy; (iv) perfect alignment (the reference case for CC). Figure 7 compares the homogenized response of the RVEs simulated for 15% stretch in tension FF. The homogenized stress-stretch curve of the RVE with perfectly aligned fibers (case iv, solid black curve) shows a far stiffer behavior compared to the RVE with isotropic fiber dispersion (case i, dotted gray curve). Intermediate cases are also significantly different.

**Table 3:** Different cases of fiber dispersion according to the shape parameters $\alpha , \beta$ of the bivariate von Mises distribution.

Case	Description	Concentration parameters	
i	In-plane and out-of-plane isotropy	$\alpha =0$,	$\beta =0$
ii	Planar isotropy and out-of-plane alignment	$\alpha =0$,	$\beta \rightarrow \infty$
iii	In-plane alignment and out-of plane isotropy	$\alpha \rightarrow \infty$,	$\beta =0$
iv	Perfect alignment	$\alpha \rightarrow \infty$,	$\beta \rightarrow \infty$

## Discussion

Overall, the model can accurately reproduce and predict the macroscale mechanical behavior of the human CC, capturing its peculiar compression-tension asymmetry^2^. In particular, the homogenized stress-stretch curve is able to replicate the nonlinearity shown by the experimental data in tension (Fig. 5b). Under compression, the model shows a certain discrepancy for the loading transverse to the fibers (Fig. 5a), but in contrast to comparable existing models^32^, it still delivers results within the experimental uncertainty. The homogenized response of the RVE shows no significant difference between different shear modes (Fig. 5c), which agrees well with experimental results^2^.

Experimental data suggest that compressing the CC along the fiber axis (compression FF) or stretching it orthogonally (tension TT) results in a softer mechanical response^2^. Since both cases involve compression of the axon fibers, the hypothesis that tortuous fibers do not bear compressive loads seems reasonable. The overall stiffness of the tissue in these two loading modes mainly results from the ground matrix^2^. A similar observation applies to the shear modes, where axon fibers are only slightly recruited at 0.2 amount of shear. In contrast, the CC is stiffer when stretched along the fibers (tension FF) or compressed orthogonally (compression TT), suggesting that recruited axon fibers provide an important contribution in these cases. In particular, tension FF shows a characteristic stiffening in the macroscopic stress-stretch curve, a feature that previous micromechanical models could not predict^31,32^.

Regarding the material parameters identified, our findings show that the axons are only slightly stiffer than the ground matrix, whereas previous studies reported that the shear modulus of the axons is several times larger^30–32^. We believe the difference is due to the simplifying assumption that axon tortuosity was neglected in previous micromechanical models. As argued by Meaney^45^, the wavy structure of axons and their distribution in tissue are critical factors that significantly affect the homogenized response of the RVE and consequently the inverse parameter identification. In addition, the fact that axons appear as soft fibers is consistent with their role being distinct from the structural function of collagen fibers^2^. We emphasize that the identified material parameters correspond to the material properties of the embedded fibers based on the modified strain-energy function. In micromechanical models that are not based on fiber embedding, the material properties of the actual axons are defined from the sum of the strain-energy functions of the ground matrix and the axon fibers.

**Figure 8:**
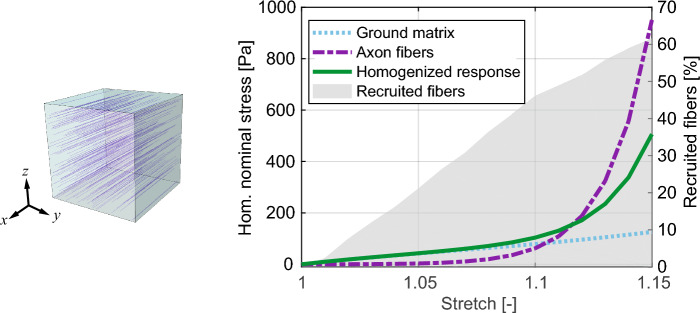
Effect of fiber recruitment. Homogenized nominal stress $\textbf{P}$ compared to the averaged stresses in the fibers ${\overline{\tilde{\textbf{P}}}}_{\textrm f}$ and in the ground matrix $\overline{\textbf{P}}_{\textrm m}$, for tension FF on the optimal RVE. The percentage of recruited fibers is also displayed.

To further highlight the effect of fiber tortuosity and recruitment, we compared the averaged stress in the fibers and in the ground matrix to the homogenized stress (Fig. 8). For stretches up to 10%, the ground matrix makes the largest contribution to the homogenized stress, whereas at higher stretches the recruited fibers contribute to the stiffening behavior. This is consistent with the experimental observations ^2^, which showed a very similar mechanical response for the CC in tension FF and tension TT at low stretches (Fig. 5b). Our model provides an explanation of such an isotropic response, based on the one hand on the reduced fraction of recruited fibers and, on the other hand, on the softness of the axon fibers. At the same time, the anisotropic response observed in other experiments with human brains at higher stretches^43^ is motivated here by the recruitment of a consistent fraction of fibers. Taken together, these observations provide a possible explanation for the long-standing debate about the mechanical anisotropy of human brain tissue. Based on our findings, we can recommend that the experimental characterization of brain tissue include a combination of various loading cases with stretches up to at least 1.15 so that a consistent number of axon fibers is recruited.

In our study we also explored the influence of axon fiber orientation with a parametric analysis. We found a very relevant effect of both in-plane and out-of-plane fiber orientation (Fig. 7), suggesting that the assumption of perfectly aligned fibers, common to previous studies on CC^30,32,33^, should be verified against new imaging data. However, it is important to note that for stretches up to 10%, the macroscale stress does not appear significantly affected by the orientation of the fibers (enlarged view of Fig. 7). This result agrees well with the observations of Budday et al. ^2^, which pointed out that there is no relevant effect of directionality in mechanical tests of CC, although the microstructure of CC has a clear structural anisotropy.

## Conclusion

We developed a model of the brain white matter based on the framework of nonlinear continuum mechanics and multiscale RVE-based numerical homogenization. The computational framework was first used to characterize the time-independent hyperelastic material properties of the microstructural constituents of white matter (ground matrix and axon fibers) using an inverse parameter identification procedure. Second, we investigated the relationship between brain microstructure and macroscopic mechanical response of the tissue. To provide a realistic and repeatable representative microstructure, a random fiber network of axons was developed based on statistical distribution functions fitted to imaging data. These include the dispersion of the axon diameter, axon fiber tortuosity and orientation. To increase the computational efficiency of the simulation, straight one-dimensional axon fibers were embedded in the volume of the ground matrix and tortuosity was included into the constitutive law through the concept of recruitment stretch.

The representative size of the volume element was determined with a convergence study performed on forty randomly generated RVEs with different sizes. By simultaneously fitting the homogenized response of the RVE to macroscale experimental data for compression and tension, the parameters of one-term Ogden models were inversely identified for both the ground matrix and the axons. To the best of the authors’ knowledge, this is the first study in which a micromechanical model of the brain is adopted to characterize the material parameters of the microstructure, including the influence of orientation and tortuosity. Incorporating the concept of fiber tortuosity and recruitment stretch, we found that axons are only slightly stiffer than the ground matrix, which is in contrast to existing similar models but in good agreement with experimental observations. Based on the identified material parameters, the simulated homogenized response showed strong agreement with the experimental data across different deformation modes. Although existing phenomenological models can fit the tension-compression response of the human brain (e.g., by adding a second term in the Ogden strain-energy function), recent microstructural models available in the literature have failed to reach this goal. In contrast, our model was able to accurately capture the response under tension based on the observed distribution of axon tortuosity without the need to introduce additional fitting parameters.

Finally, we would like to mention some limitations of the proposed model. Although it is widely accepted that brain tissue exhibits time-dependent behavior, the current study did not consider viscoelasticity or stress relaxation originating from the interaction with cerebrospinal fluid. In addition, the current study focused on a single region of the human brain white matter, and was based on the assumption of perfect fiber alignment. Existing imaging data from diffusion tensor imaging were not applicable to this study due to poor resolution at the microscopic scale of the RVE. In the future, 3D polarized light imaging could be applied to human brain tissue and potentially provide more accurate data to refine the model.

## Methods

### Axon fiber orientation

The orientation of a fiber with unit reference vector $\textbf{N}$ can be expressed with respect to a Cartesian base $\{\textbf{e}_k\}_{k=1,2,3}$ as $\textbf{N}(\Phi , \Theta )=\cos \Phi \cos \Theta \,\textbf{e}_1+\sin \Phi \cos \Theta \,\textbf{e}_2+\sin \Theta \,\textbf{e}_3$, where $\Phi \in [0, 2\pi ]$ is the azimuthal, or in-plane angle, and $\Theta \in [-\pi /2, \pi /2]$ is the elevation, or out-of-plane angle (Fig. 2d). We introduced a bivariate von Mises distribution $\rho (\Phi , \Theta )=\rho _{\textrm ip}(\Phi )\rho _{\textrm op}(\Theta )$ defined by two independent PDFs for the distribution of each angle ^54^, where

4 $$\begin{aligned} \rho _{\textrm ip}(\Phi ) = \frac{\exp \left( {\alpha }\cos 2\Phi \right) }{{I}_0({\alpha })}\,, \qquad \rho _{\textrm op}(\Theta ) = 2\sqrt{\frac{2{\beta }}{\pi }}\frac{\exp \left[ {\beta }(\cos 2\Theta -1)\right] }{{\textrm {erf}}(\sqrt{2{\beta }})}\,, \end{aligned}$$

with $I_0(\alpha )$ the modified Bessel function of the first kind of order zero and ${\textrm {erf}}(\bullet )$ the error function of $(\bullet )$.

### Recruitment stretch

To describe the tortuosity of axon fibers, we defined a straightness parameter $P_{\textrm s} = {L_0}/{L_{\textrm f}}$ and employed it in the concept of recruitment stretch. Assuming a multiplicative decomposition of the kinematic stretch $\lambda$ in a single straight fiber according to $\lambda =\frac{l}{L_{\textrm 0}} = \lambda _{\textrm r} \lambda _{\textrm t}$, we can distinguish the recruitment stretch $\lambda _{\textrm r}$ and the true stretch $\lambda _{\textrm t}$^58^. In the previous equation, *l* is the deformed length of the straight fiber (see Fig. 2c). The value of $\lambda _{\textrm t}$ depends on the state of the fiber, i.e.

5 $$\begin{aligned} {\left\{ \begin{array}{ll} \lambda _{\textrm r} = \lambda , \quad \lambda _{\textrm t} = 1, &{} \hspace{1cm}\text {if}\hspace{1cm} 1\le \lambda <\dfrac{1}{P_{\textrm s}}\,,\\ \lambda _{\textrm r} = \dfrac{1}{P_{\textrm s}}, \quad \lambda _{\textrm t} = \dfrac{l}{L_{\textrm f}} = \lambda P_{{\textrm s}}, &{} \hspace{1cm}\text {if}\hspace{1cm} \lambda \ge \dfrac{1}{P_{\textrm s}} \,. \end{array}\right. } \end{aligned}$$

### Material models

The ground matrix behaves like a nearly incompressible material whose strain-energy function is expressed as an isotropic one-term Ogden material model, i.e.

6 $$\begin{aligned} \Psi _{\textrm m}=\frac{2\mu _{\textrm m}}{\alpha _{\textrm m}^2}(\lambda _1^{\alpha _{\textrm m}}+\lambda _2^{\alpha _{\textrm m}}+\lambda _3^{\alpha _{\textrm m}}-3) + \frac{1}{D_{\textrm m}}(J-1)^2\,, \end{aligned}$$

where $\lambda _k$, $k = 1,2,3$, are the principal stretches, and $J = \lambda _1 \lambda _2 \lambda _3$ is the volume ratio. To enforce near incompressibility, we assumed the volumetric parameter $D_{\textrm m} = 0.04/\mu _{\textrm m}$, which corresponds to an initial Poisson’s ratio of $\nu _{\textrm m} = 0.49$. The strain-energy function for the *i*-th embedded fiber includes the concept of recruitment stretch and is defined as

7 $$\begin{aligned} \tilde{\Psi }_{\textrm f}^{(i)}= \dfrac{2\mu _{\textrm f}}{\alpha _{\textrm f}^2}(\lambda _{\textrm t}^{\alpha _{\textrm f}} + 2\lambda _{\textrm t}^{-\alpha _{\textrm f}/2} - 3)\,, \end{aligned}$$

with the true stretch $\lambda _{\textrm t}$ according to (5).

### Finite element model

#### Embedded element technique

The microscopic RVE, subjected to the elementary deformation modes, was solved using the finite element solver of Abaqus FEA software (Dassault Systèmes) using the embedded element technique. In the embedded element technique, a guest domain (axon fibers) is superposed on a host domain (ground matrix). The formulation implemented in Abaqus FEA constrains the translational degrees of freedom of the embedded nodes to the elements of the surrounding matrix. In our model, the ground matrix is meshed by first-order hexahedral mixed-formulation elements (C3D8H) and the fibers are meshed by first-order mixed-formulation truss elements (T3D2H). To ensure the condition of no-slip between the matrix and fibers, the fibers are carefully meshed so that the embedded nodes lie precisely on the edge or face of the host elements. A mesh convergence analysis for the RVE with $L_{\textrm a}=25~\mu$m was performed, considering the following element sizes of 2.50, 1.25, 0.83 and $0.75\,\mu$m for both 3D and 1D elements. The results showed that the homogenized response of the RVEs is independent of the element size across all loading modes. An element size of $1.25\,\mu$m was adopted in all simulations.

The embedded element technique creates a stiffness redundancy for the matrix volume occupied by the fibers. Various approaches are discussed in the literature to address the stiffness redundancy^19,30–32^. Based on the rule of mixtures and the no-slip condition, we can write the total energy *W* stored in the continuum medium as

8 $$\begin{aligned} W&=\int _{V_{\textrm m}}\Psi _{\textrm m}{\textrm d}V + \sum _{i=1}^{N_{\textrm f}}\int _{V_{\textrm f}^{(i)}}\tilde{\Psi }_{\textrm f}^{(i)}{\textrm d}V\,, \end{aligned}$$

where $\tilde{\Psi }_{\textrm f}^{(i)}=\Psi _{\textrm f}^{(i)}-\Psi _{\textrm m}$ denotes the modified strain-energy function of the *i*-th embedded fiber and $N_{\textrm{f}}$ is the total number of fibers.

#### Multiscale boundary conditions

In the multiscale theory based on RVEs and stress homogenization, periodic boundary conditions are applied to ensure that opposite faces at the boundary of the RVE deform identically. Mathematically, we require that each pair of points $(\textbf{X}^+,\textbf{X}^-)$ on opposite surfaces of the boundary $\partial \Omega _0 = \partial \Omega _0^+ \cup \partial \Omega _0^-$ has the same displacement fluctuation $\tilde{\textbf{u}}$, i.e.

9 $$\begin{aligned} \textbf{u}&= (\textbf{F} - \textbf{I})\textbf{X} + \tilde{\textbf{u}}, \qquad \forall \textbf{X} \in \partial \Omega _0\,, \end{aligned}$$

10 $$\begin{aligned} \tilde{\textbf{u}}(\textbf{X}^+)&= \tilde{\textbf{u}}(\textbf{X}^-), \qquad \forall \textbf{X}^+ \in \partial \Omega _0^+,\, \forall \textbf{X}^- \in \partial \Omega _0^-\,, \end{aligned}$$

where $\textbf{F}$ is the macroscopic deformation gradient and $\textbf{I}$ is the second-order identity tensor. The resulting set of linear constraint equations correlates the displacement degrees of freedom of the node pairs on opposite faces, edges, and corners of the RVE^59^. The constraints are implemented using a custom Python script and are provided in detail in the Supplementary Material available online.

#### Inverse parameter identification

We devised a two-step optimization algorithm to characterize the mechanical parameters of the ground matrix and the embedded axon fibers based on experimental data^2^. First, the ground matrix parameters $\mu _{\textrm m}$ and $\alpha _{\textrm m}$ are determined from compression FF and tension TT up to 10% stretch, fitting the experimental data to the analytical stress-stretch curves in the incompressible limit at the same time. Afterward, the parameters $\mu _{\textrm f}$ and $\alpha _{\textrm f}$ of the embedded fibers are obtained by an inverse procedure based on finite element simulations on the RVE and experimental data in tension FF up to 10% stretch. The use of analytical curves in part of the parameter identification significantly reduced the computational time. The boundaries for the material constants were set to $\mu _{\textrm m} \in [50, 5\,000]$ Pa, $\alpha _{\textrm m} \in [-50, 50]$, $\mu _{\textrm f} \in [20, 5000]$ Pa and $\alpha _{\textrm f} \in [-{\textrm {inf}}, {\textrm {inf}}]$. Based on an initial guess of the material parameters, the optimization algorithm minimizes the absolute squared error between the experimental data and the simulation output data according to the maximum likelihood principle, i.e. ${\chi}^2 = \sum _{n=1}^N ({P}_{ij}^{{\textrm {exp}}}-{P}_{ij}^{{\textrm {sim}}})_n^2$, where ${P}_{ij}^{{\textrm {exp}}}$ and ${P}_{ij}^{{\textrm {sim}}}$ are the components of the nominal stress tensor from the experiments and the analytical or finite element simulation, respectively.

The process of identifying the material parameters was performed using a custom Python script integrated into the Abaqus FEA software. A trust–region reflective algorithm was employed^60^. In order to improve the computational efficiency of the finite element analyses, the simulations and post-processing were parallelized. Sensitivity analyses were carried out to verify that the optimal values are independent of the initial guesses and the boundary values. The algorithm is summarized in the Supplementary Material available online.

### Supplementary Information

Supplementary Information 1.

## Data Availability

Algorithms used for generating the custom codes during the current study are included in the Supplementary Material. All of the requested data for the 3D models and custom codes are available from the corresponding author on request.

## References

[1] Prange MT, Margulies SS (2002). Regional, Directional, and Age-Dependent Properties of the Brain Undergoing Large Deformation. J. Biomech. Eng..

[2] Budday S (2017). Mechanical characterization of human brain tissue. Acta Biomater..

[3] Budday S, Ovaert TC, Holzapfel GA, Steinmann P, Kuhl E (2020). Fifty shades of brain: A review on the mechanical testing and modeling of brain tissue. Arch. Comput. Methods Eng..

[4] Goriely A (2015). Mechanics of the brain: perspectives, challenges, and opportunities. Biomech. Model. Mechanobiol..

[5] Weickenmeier J (2016). Brain stiffness increases with myelin content. Acta Biomater..

[6] Weickenmeier J, de Rooij R, Budday S, Ovaert TC, Kuhl E (2017). The mechanical importance of myelination in the central nervous system. J. Mech. Behav. Biomed. Mater..

[7] Weickenmeier J, Jucker M, Goriely A, Kuhl E (2019). A physics-based model explains the prion-like features of neurodegeneration in Alzheimer’s disease, Parkinson’s disease, and amyotrophic lateral sclerosis. J. Mech. Phys. Solids.

[8] Blinkouskaya Y, Caçoilo A, Gollamudi T, Jalalian S, Weickenmeier J (2021). Brain aging mechanisms with mechanical manifestations. Mech. Ageing Dev..

[9] Reiter N, Paulsen F, Budday S (2023). Mechanisms of mechanical load transfer through brain tissue. Sci. Rep..

[10] Kilinc D, Blasiak A, Lee GU (2015). Microtechnologies for studying the role of mechanics in axon growth and guidance. Front. Cell. Neurosci..

[11] Koser DE (2016). Mechanosensing is critical for axon growth in the developing brain. Nat. Neurosci..

[12] Wang LM, Kuhl E (2023). Mechanics of axon growth and damage: A systematic review of computational models. Semin. Cell Dev. Biol..

[13] Lanir Y (1979). A structural theory for the homogeneous biaxial stress-strain relationships in flat collagenous tissues. J. Biomech..

[14] Gasser TC, Ogden RW, Holzapfel GA (2006). Hyperelastic modelling of arterial layers with distributed collagen fibre orientations. J. R. Soc. Interface.

[15] Li K, Ogden RW, Holzapfel GA (2018). A discrete fibre dispersion method for excluding fibres under compression in the modelling of fibrous tissues. J. R. Soc. Interface.

[16] Carniel TA, Fancello EA (2019). A variational homogenization approach applied to the multiscale analysis of the viscoelastic behavior of tendon fascicles. Continuum Mech. Thermodyn..

[17] Stylianopoulos T, Barocas VH (2007). Multiscale, structure-based modeling for the elastic mechanical behavior of arterial walls. J. Biomech. Eng..

[18] Shah SB (2014). Prefailure and failure mechanics of the porcine ascending thoracic aorta: experiments and a multiscale model. J. Biomech. Eng..

[19] Dalbosco M, Carniel TA, Fancello EA, Holzapfel GA (2021). Multiscale numerical analyses of arterial tissue with embedded elements in the finite strain regime. Comput. Methods Appl. Mech. Eng..

[20] Li DS, Mendiola EA, Avazmohammadi R, Sachse FB, Sacks MS (2023). A multi-scale computational model for the passive mechanical behavior of right ventricular myocardium. J. Mech. Behav. Biomed. Mater..

[21] de Souza Neto EA, Blanco PJ, Sánchez PJ, Feijóo RA (2015). An RVE-based multiscale theory of solids with micro-scale inertia and body force effects. Mech. Mater..

[22] Blanco PJ, Sánchez PJ, de Souza Neto EA, Feijóo RA (2016). Variational foundations and generalized unified theory of RVE-based multiscale models. Arch. Comput. Methods Eng..

[23] Arbogast KB, Margulies SS (1999). A fiber-reinforced composite model of the viscoelastic behavior of the brainstem in shear. J. Biomech..

[24] Karami G, Grundman N, Abolfathi N, Naik A, Ziejewski M (2009). A micromechanical hyperelastic modeling of brain white matter under large deformation. J. Mech. Behav. Biomed. Mater..

[25] Cloots RJH, van Dommelen JAW, Nyberg T, Kleiven S, Geers MGD (2011). Micromechanics of diffuse axonal injury: influence of axonal orientation and anisotropy. Biomech. Model. Mechanobiol..

[26] Pan Y, Sullivan D, Shreiber DI, Pelegri AA (2013). Finite element modeling of CNS white matter kinematics: use of a 3D RVE to determine material properties. Front. Bioengi. Biotechnol..

[27] Javid S, Rezaei A, Karami G (2014). A micromechanical procedure for viscoelastic characterization of the axons and ECM of the brainstem. J. Mech. Behav. Biomed. Mater..

[28] Zarei V, Zhang S, Winkelstein BA, Barocas VH (2017). Tissue loading and microstructure regulate the deformation of embedded nerve fibres: predictions from single-scale and multiscale simulations. J. R. Soc. Interface.

[29] Montanino A, Saeedimasine M, Villa A, Kleiven S (2019). Axons embedded in a tissue may withstand larger deformations than isolated axons before mechanoporation occurs. J. Biomech. Eng..

[30] Yousefsani SA, Farahmand F, Shamloo A (2018). A three-dimensional micromechanical model of brain white matter with histology-informed probabilistic distribution of axonal fibers. J. Mech. Behav. Biomed. Mater..

[31] Hoursan H, Farahmand F, Ahmadian MT (2020). A three-dimensional statistical volume element for histology informed micromechanical modeling of brain white matter. Ann. Biomed. Eng..

[32] Chavoshnejad P, German GK, Razavi MJ (2021). Hyperelastic material properties of axonal fibers in brain white matter. Brain Multiphys..

[33] Jamal A, Bernardini A, Dini D (2022). Microscale characterisation of the time-dependent mechanical behaviour of brain white matter. J. Mech. Behav. Biomed. Mater..

[34] Ronen I (2014). Microstructural organization of axons in the human corpus callosum quantified by diffusion-weighted magnetic resonance spectroscopy of N-acetylaspartate and post-mortem histology. Brain Struct. Funct..

[35] Menzel M (2020). Toward a high-resolution reconstruction of 3D nerve fiber architectures and crossings in the brain using light scattering measurements and finite-difference time-domain simulations. Phys. Rev. X.

[36] Yousefsani SA, Karimi MZV (2023). Bidirectional hyperelastic characterization of brain white matter tissue. Biomech. Model. Mechanobiol..

[37] Donnelly BR, Medige J (1997). Shear Properties of Human Brain Tissue. J. Biomech. Eng..

[38] Franceschini G, Bigoni D, Regitnig P, Holzapfel GA (2006). Brain tissue deforms similarly to filled elastomers and follows consolidation theory. J. Mech. Phys. Solids.

[39] Forte AE, Gentleman SM, Dini D (2017). On the characterization of the heterogeneous mechanical response of human brain tissue. Biomech. Model. Mechanobiol..

[40] Budday S, Sommer G, Holzapfel G, Steinmann P, Kuhl E (2017). Viscoelastic parameter identification of human brain tissue. J. Mech. Behav. Biomed. Mater..

[41] Lebel C (2012). Diffusion tensor imaging of white matter tract evolution over the lifespan. Neuroimage.

[42] Anderson AT (2016). Observation of direction-dependent mechanical properties in the human brain with multi-excitation MR elastography. J. Mech. Behav. Biomed. Mater..

[43] Velardi F, Fraternali F, Angelillo M (2006). Anisotropic constitutive equations and experimental tensile behavior of brain tissue. Biomech. Model. Mechanobiol..

[44] Feng Y, Okamoto RJ, Namani R, Genin GM, Bayly PV (2013). Measurements of mechanical anisotropy in brain tissue and implications for transversely isotropic material models of white matter. J. Mech. Behav. Biomed. Mater..

[45] Meaney DF (2003). Relationship between structural modeling and hyperelastic material behavior: application to CNS white matter. Biomech. Model. Mechanobiol..

[46] Ramzanpour M, Hosseini-Farid M, McLean J, Ziejewski M, Karami G (2020). Visco-hyperelastic characterization of human brain white matter micro-level constituents in different strain rates. Med. Biol. Eng. Compu..

[47] Zemmoura I (2016). How Klingler’s dissection permits exploration of brain structural connectivity? An electron microscopy study of human white matter. Brain Struct. Funct..

[48] Bernardini A (2022). Reconstruction of ovine axonal cytoarchitecture enables more accurate models of brain biomechanics. Commun. Biol..

[49] Yuan T, Zhan W, Dini D (2023). Linking fluid-axons interactions to the macroscopic fluid transport properties of the brain. Acta Biomater..

[50] Garimella HT, Menghani RR, Gerber JI, Sridhar S, Kraft RH (2019). Embedded finite elements for modeling axonal injury. Ann. Biomed. Eng..

[51] Rezakhaniha R (2012). Experimental investigation of collagen waviness and orientation in the arterial adventitia using confocal laser scanning microscopy. Biomech. Model. Mechanobiol..

[52] Liewald D, Miller R, Logothetis N, Wagner H-J, Schüz A (2014). Distribution of axon diameters in cortical white matter: an electron-microscopic study on three human brains and a macaque. Biol. Cybern..

[53] Sepehrband F (2016). Parametric probability distribution functions for axon diameters of corpus callosum. Front. Neuroanat..

[54] Holzapfel GA, Niestrawska JA, Ogden RW, Reinisch AJ, Schriefl AJ (2015). Modelling non-symmetric collagen fibre dispersion in arterial walls. J. R. Soc. Interface.

[55] Nilsson M, Lätt J, Ståhlberg F, van Westen D, Hagslätt H (2012). The importance of axonal undulation in diffusion MR measurements: A Monte Carlo simulation study. NMR Biomed..

[56] Schilling KG (2018). Histological validation of diffusion MRI fiber orientation distributions and dispersion. Neuroimage.

[57] Valdés Cabrera D (2023). High-resolution diffusion tensor imaging and T2 mapping detect regional changes within the hippocampus in multiple sclerosis. NMR in Biomed..

[58] Lu J, He X (2021). Incorporating fiber recruitment in hyperelastic modeling of vascular tissues by means of kinematic average. Biomech. Model. Mechanobiol..

[59] Tian W, Qi L, Chao X, Liang J, Fu M (2019). Periodic boundary condition and its numerical implementation algorithm for the evaluation of effective mechanical properties of the composites with complicated micro-structures. Compos. B Eng..

[60] Hinrichsen J (2023). Inverse identification of region-specific hyperelastic material parameters for human brain tissue. Biomech. Model. Mechanobiol..

