# Supplementary material for: Histology-informed multiscale modeling of human brain white matter
Source: Sci Rep. 2023 Nov 10;13:19641. doi: 10.1038/s41598-023-46600-3 (PMC10638412; doi:10.1038/s41598-023-46600-3)
Supplement: Supplementary file 1 — Supplementary Information 1. [file 41598_2023_46600_MOESM1_ESM.pdf]

Histology-informed multiscale modeling of human brain white matter

S. Saeidi, M.P. Kainz, M. Dalbosco, M. Terzano, G.A. Holzapfel

Supplementary Material

Supplementary Material to this paper provides additional details to better understand the modeling approach and its numerical implementation.

3D fiber generation algorithm

According to the approach described in Dalbosco et al.[1], the 3D representative volume element (RVE) of the human brain white matter consists of a cube representing the brain ground matrix, with a randomly generated network of axon fibers modeled by 1D truss elements.

The procedure starts by taking an individual fiber with diameter $d^{(i)}$, straightness parameter $P_\mathrm{s}^{(i)}$, in-plane and out-of-plane angles $\Phi^{(i)}$ and $\Theta^{(i)}$ assigned by randomizing a value from the probability density functions (PDFs), see Eqs. (1),(2),(4) in the paper. Then the RVE edge length L_a and the volume fraction of the axon fibers v_f are introduced. The axon volume fraction v_f is the ratio between the volume of axons and the total volume of the RVE, i.e.

$$v_\mathrm{f} = \frac{\sum_{i=1}^{N_\mathrm{f}} V_\mathrm{f}^{(i)}}{V_\mathrm{m}} = \frac{\sum_{i=1}^{N_\mathrm{f}} \pi {d^{(i)}}^2 L_\mathrm{f}^{(i)}}{4L_\mathrm{a}^3}, \tag{S1}$$

where V_m is the volume of the ground matrix, $V_\mathrm{f}^{(i)}$ is the volume of the i-th fiber, $L_\mathrm{f}^{(i)}$ refers to its arc-length and N_f is the total number of fibers. The human corpus callosum has a dense network of axon fibers that make up about 40% of its total volume[2].

Within the cube, a random nucleation point is chosen and the direction of the i-th fiber is determined as $\mathbf{N}(\Phi^{(i)}, \Theta^{(i)})$. The i-th fiber center line is then extended along the predefined direction until it crosses the boundaries of the cube and the coordinates of the two endpoints $(\mathbf{X}^{1(i)}, \mathbf{X}^{2(i)})$ are used to determine its end-to-end length $L_0^{(i)}$. Based on the values of $P_\mathrm{s}^{(i)}$ and $d^{(i)}$ the volume fraction $v_\mathrm{f}^{(i)}$ is then calculated. The process is repeated until the target volume fraction v_f is reached. The fiber generation algorithm is implemented directly in the finite element (FE) analysis software Abaqus FEA 2018[3] via a custom Python code. Algorithm S1 summarizes the whole procedure.

Algorithm S1 Generation of the random axon fiber network

Require: $L_\mathrm{a}, \mu, \sigma, \xi, a, b, \alpha, \beta, v_\mathrm{f}, \mathrm{d}L$
  ▷ *Initializing*
  $V_\mathrm{f} = 0$
  $v_\mathrm{f}^\mathrm{curr} = 0$
  $i = 1$
  while $v_\mathrm{f}^{curr} < v_\mathrm{f}$ **do**
  ▷ *Sampling parameters from PDFs*
    Sample diameter $d^{(i)}$ ▷ See Eq. (1)
    Sample in-plane angle $\Phi^{(i)}$ ▷ See Eq. (4)
    Sample out-of-plane angle $\Theta^{(i)}$ ▷ See Eq. (4)
    Calculate fiber unit vector $\mathbf{N}(\Phi^{(i)}, \Theta^{(i)})$
    Sample $P_\mathrm{s}^{(i)}$ ▷ See Eq. (2)
  ▷ *Creating random fiber*
    Create nucleation point $[\mathbf{X}^0] = [X_1^0, X_2^0, X_3^0]$, with $X_k^0 \in [0, \mathrm{L_a}]$, $k = 1, 2, 3$
    $X_k^1 = X_k^0$
    while $X_k^1 \in [0, \mathrm{L_a}]$ **do** ▷ 1st vertex of fiber
      $\mathbf{X}^1 = \mathbf{X}^1 - \mathrm{d}L\mathbf{N}$
    end while
    $\mathbf{X}^{1(i)} = \mathbf{X}^1$
    $X_k^2 = X_k^0$
    while $X_k^2 \in [0, \mathrm{L_a}]$ **do** ▷ 2nd vertex of fiber
      $\mathbf{X}^2 = \mathbf{X}^2 + \mathrm{d}L\mathbf{N}$
    end while
    $\mathbf{X}^{2(i)} = \mathbf{X}^2$
  ▷ *Computing fiber volume fraction*
    $L_0^{(i)} = ||\mathbf{X}^{1(i)} - \mathbf{X}^{2(i)}||$
    $L_\mathrm{f}^{(i)} = L_0^{(i)} / P_\mathrm{s}^{(i)}$
    $V_\mathrm{f} = V_\mathrm{f} + \pi {d^{(i)}}^2 L_\mathrm{f}^{(i)} / 4$
    $v_\mathrm{f}^\mathrm{curr} = V_\mathrm{f} / \mathrm{L_a^3}$ ▷ See Eq. (S1)
    $i = i + 1, N_\mathrm{f} = i$
  end while
  return $N_\mathrm{f}, \mathbf{X}^{1(i)}, \mathbf{X}^{2(i)}, d^{(i)}, \Phi^{(i)}, \Theta^{(i)}, P_\mathrm{s}^{(i)}, \forall i \in [1, N_\mathrm{f}]$

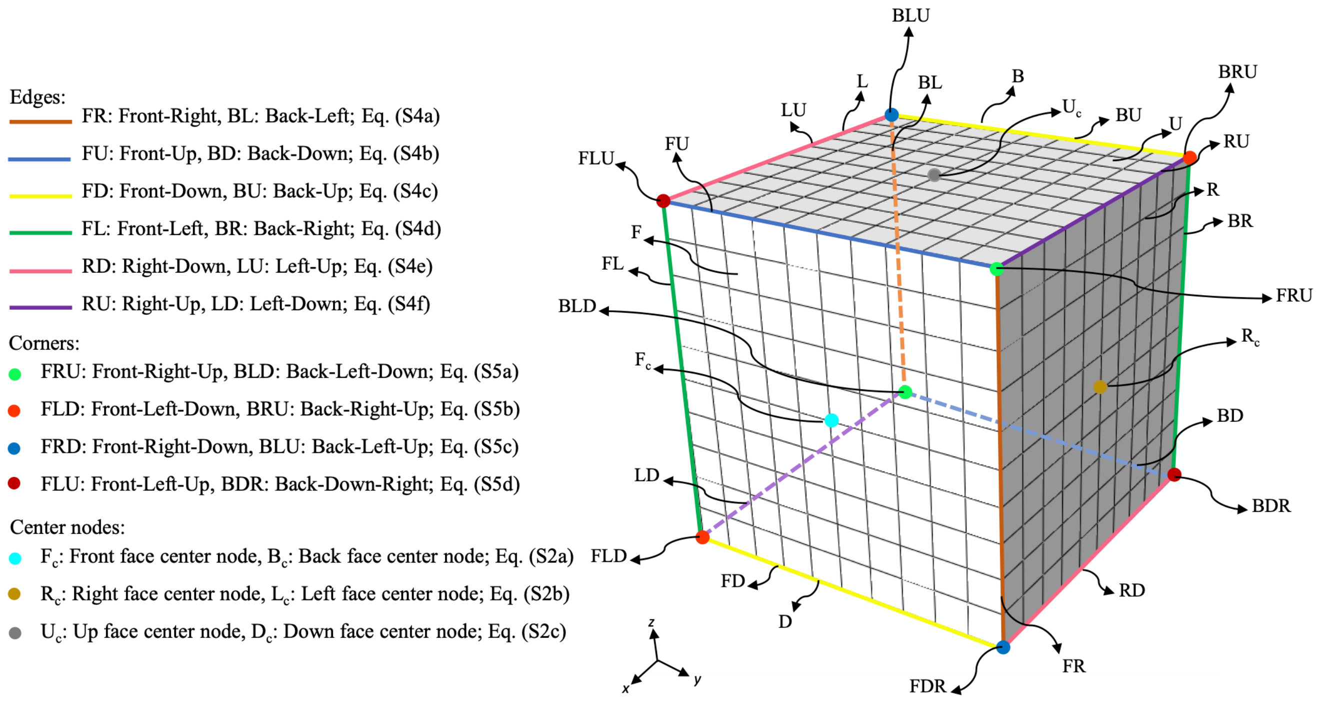

Figure S1. Visualization of the RVE with the notation used for opposite faces, edges and corners. Front face (F), Right face (R), Upper face (U), Back face (B), Left face (L), Lower face (D) and a combination of these superscripts for referring to edges and corners. Additional subscript c for the center nodes on each face.

Periodic boundary conditions

In the following, we report the periodic boundary conditions applied to the RVE of the brain white matter with respect to the macroscopic deformations defined in the main article.

Each RVE contains 6 faces, 12 edges, and 8 corner nodes (shown in Figure S1). The front (F), right (R) and upper (U) faces are considered as master faces with their associated slave faces back (B), left (L) and lower (D). Edges and corners are marked with superscripts of the intersecting faces. The center nodes (additional superscript c) on opposite faces follow the same displacement. Symmetric mesh is required on opposite faces of the RVE. The linear constraint equations provided below correlate the displacement of nodes on master faces, edges, and corners with respect to their associated node pairs on slave faces, edges and corners. The displacement degrees of freedom at each node are defined as $u_k, k = 1, 2, 3$.

Center nodes:

$$u_k^{\mathrm{F_c}} + u_k^{\mathrm{B_c}} = 0\,, \tag{S2a}$$

$$u_k^{\mathrm{R_c}} + u_k^{\mathrm{L_c}} = 0\,, \tag{S2b}$$

$$u_k^{\mathrm{U_c}} + u_k^{\mathrm{D_c}} = 0\,. \tag{S2c}$$

Faces:

$$u_k^{\mathrm{F}} - u_k^{\mathrm{B}} - 2u_k^{\mathrm{F_c}} = 0\,, \tag{S3a}$$

$$u_k^{\mathrm{R}} - u_k^{\mathrm{L}} - 2u_k^{\mathrm{R_c}} = 0\,, \tag{S3b}$$

$$u_k^{\mathrm{U}} - u_k^{\mathrm{D}} - 2u_k^{\mathrm{U_c}} = 0\,. \tag{S3c}$$

Edges:

$$u_k^{\mathrm{FR}} - u_k^{\mathrm{BL}} - 2u_k^{\mathrm{F_c}} - 2u_k^{\mathrm{R_c}} = 0\,, \tag{S4a}$$

$$u_k^{\mathrm{FU}} - u_k^{\mathrm{BD}} - 2u_k^{\mathrm{F_c}} - 2u_k^{\mathrm{U_c}} = 0\,, \tag{S4b}$$

$$u_k^{\mathrm{FD}} - u_k^{\mathrm{BU}} - 2u_k^{\mathrm{F_c}} + 2u_k^{\mathrm{U_c}} = 0\,, \tag{S4c}$$

$$u_k^{\mathrm{FL}} - u_k^{\mathrm{BR}} + 2u_k^{\mathrm{R_c}} - 2u_k^{\mathrm{F_c}} = 0\,, \tag{S4d}$$

$$u_k^{\mathrm{RD}} - u_k^{\mathrm{LU}} + 2u_k^{\mathrm{U_c}} - 2u_k^{\mathrm{R_c}} = 0\,, \tag{S4e}$$

$$u_k^{\mathrm{RU}} - u_k^{\mathrm{LD}} - 2u_k^{\mathrm{U_c}} - 2u_k^{\mathrm{R_c}} = 0\,. \tag{S4f}$$

Corners:

$$u_k^{\mathrm{FRU}} - u_k^{\mathrm{BLD}} - 2(u_k^{\mathrm{F_c}} + u_k^{\mathrm{R_c}} + u_k^{\mathrm{U_c}}) = 0\,, \tag{S5a}$$

$$u_k^{\mathrm{FLD}} - u_k^{\mathrm{BRU}} + 2(u_k^{\mathrm{U_c}} + u_k^{\mathrm{R_c}} - u_k^{\mathrm{F_c}}) = 0\,, \tag{S5b}$$

$$u_k^{\mathrm{FRD}} - u_k^{\mathrm{BLU}} - 2(u_k^{\mathrm{F_c}} + u_k^{\mathrm{R_c}} - u_k^{\mathrm{U_c}}) = 0\,, \tag{S5c}$$

$$u_k^{\mathrm{FLU}} - u_k^{\mathrm{BDR}} - 2(u_k^{\mathrm{F_c}} + u_k^{\mathrm{U_c}} - u_k^{\mathrm{R_c}}) = 0\,. \tag{S5d}$$

In addition, the three center nodes are used on master faces to apply the different loading conditions. Specifically,

we have

$$\text{Tension/compression FF:} \qquad u_1^{\mathrm{F_c}} = \pm(\lambda_1 - 1)L_\mathrm{a}/2\,, \tag{S6a}$$

$$\text{Tension/compression TT:} \qquad u_2^{\mathrm{R_c}} = \pm(\lambda_2 - 1)L_\mathrm{a}/2\,, \tag{S6b}$$

$$\text{Shear TF:} \qquad u_1^{\mathrm{U_c}} = \pm\gamma_{12}/(2L_\mathrm{a})\,, \tag{S6c}$$

$$\text{Shear TT:} \qquad u_2^{\mathrm{U_c}} = \pm\gamma_{21}/(2L_\mathrm{a})\,, \tag{S6d}$$

$$\text{Shear FT:} \qquad u_3^{\mathrm{U_c}} = \pm\gamma_{31}/(2L_\mathrm{a})\,, \tag{S6e}$$

where λ_1, λ_2 are the prescribed principal stretches along directions parallel to the x-axis and y-axis, respectively, while $\gamma_{12}, \gamma_{21}, \gamma_{31}$ denote the amount of prescribed shear.

Additional boundary conditions are applied to restrict the rigid body motion of the RVE. For all loading scenarios, one node is fixed in the center of the RVE and the center node on edge FU is fixed in direction y to prevent rotation around the x-axis. In addition, the following constraints are applied:

$$\text{Tension/Compression FF:} \qquad u_2^{\mathrm{F_c}} = 0\,, u_3^{\mathrm{F_c}} = 0\,, \tag{S7a}$$

$$\text{Tension/Compression TT:} \qquad u_1^{\mathrm{R_c}} = 0\,, u_3^{\mathrm{R_c}} = 0\,, \tag{S7b}$$

$$\text{Shear TF:} \qquad u_3^{\mathrm{U_c}} = 0\,, \tag{S7c}$$

$$\text{Shear TT:} \qquad u_3^{\mathrm{U_c}} = 0\,, \tag{S7d}$$

$$\text{Shear FT:} \qquad u_1^{\mathrm{F_c}} = 0\,. \tag{S7e}$$

Homogenized stress computation

The homogenized first Piola–Kirchhoff stress tensor is calculated according to $\mathbf{P} = \overline{\mathbf{P}}_\mathrm{m} + v_\mathrm{f}\overline{\tilde{\mathbf{P}}}_\mathrm{f}$. First, the average stress tensors in the ground matrix $\overline{\mathbf{P}}_\mathrm{m}$ and in the embedded fibers $\overline{\tilde{\mathbf{P}}}_\mathrm{f}$ are calculated based on the concept of volume-averaging as follows

$$\overline{\mathbf{P}}_\mathrm{m} = \frac{1}{V_\mathrm{m}} \int_{V_\mathrm{m}} \mathbf{P}_\mathrm{m} \mathrm{d}V\,, \qquad \overline{\tilde{\mathbf{P}}}_\mathrm{f} = \frac{1}{V_\mathrm{f}} \sum_{i=1}^{N_\mathrm{f}} \int_{V_\mathrm{f}^{(i)}} \tilde{\mathbf{P}}_\mathrm{f}^{(i)} \mathrm{d}V\,, \tag{S8}$$

where $\mathbf{P}_\mathrm{m}$ and $\tilde{\mathbf{P}}_\mathrm{f}^{(i)}$ can be derived from the respective strain-energy functions as

$$\mathbf{P}_\mathrm{m} = \sum_{k=1}^{3} \frac{\partial \Psi_\mathrm{m}}{\partial \lambda_k} \mathbf{n}_k \otimes \mathbf{N}_k - p\mathbf{F}^{-\mathrm{T}}\,, \quad \tilde{\mathbf{P}}_\mathrm{f}^{(i)} = \frac{\partial \tilde{\Psi}_\mathrm{f}^{(i)}}{\partial \lambda_1} \mathbf{n}_1 \otimes \mathbf{N}_1 - p\lambda_1^{-1}\,, \tag{S9}$$

with $\mathbf{n}_k$ and $\mathbf{N}_k$ the eigenvectors of the left and right Cauchy-Green tensors and λ_k the square root of their eigenvalues[4].

At this point we recall that the strain energy of the fibers has been corrected to take into account the stiffness redundancy associated with the use of embedded elements. Based on the rule of mixtures, the total strain energy of the RVE can be expressed as

$$W = \int_{V_\mathrm{m}/V_\mathrm{f}} \Psi_\mathrm{m} \mathrm{d}V + \sum_{i=1}^{N_f} \int_{V_\mathrm{f}^{(i)}} \Psi_\mathrm{f}^{(i)} \mathrm{d}V \,, \tag{S10}$$

where $\Psi_\mathrm{f}^{(i)}$ is the strain-energy function of the 'actual' i-th fiber. Based on the no-slip assumption, the matrix and embedded fibers continuously deform; therefore, the stored energy of the ground matrix can be decomposed into two parts, i.e.

$$W = \int_{V_\mathrm{m}} \Psi_\mathrm{m} \mathrm{d}V - \sum_{i=1}^{N_\mathrm{f}} \int_{V_\mathrm{f}^{(i)}} \Psi_\mathrm{m} \mathrm{d}V + \sum_{i=1}^{N_\mathrm{f}} \int_{V_\mathrm{f}^{(i)}} \Psi_\mathrm{f}^{(i)} \mathrm{d}V = \int_{V_\mathrm{m}} \Psi_\mathrm{m} \mathrm{d}V + \sum_{i=1}^{N_\mathrm{f}} \int_{V_\mathrm{f}^{(i)}} \tilde{\Psi}_\mathrm{f}^{(i)} \mathrm{d}V \,, \tag{S11}$$

where $\tilde{\Psi}_\mathrm{f}^{(i)} = \Psi_\mathrm{f}^{(i)} - \Psi_\mathrm{m}$ denotes the modified strain-energy function of the i-th embedded fiber appearing in Eq. (S9).

In our implementation for Abaqus FEA, we derive the first Piola–Kirchhoff stress tensors in the ground matrix and embedded fibers using pull-back operations on the Cauchy stress tensors, given as $\mathbf{P} = J\boldsymbol{\sigma}\mathbf{F}^{-\mathrm{T}}$[4]. Preliminarily, we had to transform the Cauchy stress tensors expressed in the local coordinate systems of the elements to the global system. In our model, fibers are embedded in the host mesh of the ground matrix. Therefore, we can distinguish three coordinate systems: the global coordinate system, in which the host and embedded meshes are defined, with basis $\{\mathbf{e}_k\}_{k=1,2,3}$; a local coordinate system of the host element, in which the respective stress tensor is defined, with basis $\{\mathbf{g}_k\}_{k=1,2,3}$; a local coordinate system of the embedded truss element, with basis $\{\mathbf{v}_k\}_{k=1,2,3}$, where $\mathbf{v}_1$ is the fiber axis[5]. The stress tensor in the truss elements has only one component, which is defined in the local coordinate system of the embedded truss element along the fiber axis $\mathbf{v}_1$. Because the basis of the local coordinate system of the host element is aligned with the basis of the global coordinate system, we just need to transform the stress tensor in the truss elements to the global coordinate system, using the following matrix transformation

$$[\boldsymbol{\sigma}]^{(\mathbf{e})} = [\mathbf{T}][\boldsymbol{\sigma}]^{(\mathbf{v})}[\mathbf{T}]^\mathrm{T} \,, \tag{S12}$$

where $[\boldsymbol{\sigma}]^{(\mathbf{v})}$ and $[\boldsymbol{\sigma}]^{(\mathbf{e})}$ are the Cauchy stress matrices in the local coordinate system of the embedded truss element

and in the global coordinate system, respectively, and the transformation matrix $[\mathbf{T}]$ is defined as

$$[\mathbf{T}] = \begin{bmatrix} \mathbf{v}_1 \cdot \mathbf{e}_1 & \mathbf{v}_1 \cdot \mathbf{e}_2 & \mathbf{v}_1 \cdot \mathbf{e}_3 \\ \mathbf{v}_2 \cdot \mathbf{e}_1 & \mathbf{v}_2 \cdot \mathbf{e}_2 & \mathbf{v}_2 \cdot \mathbf{e}_3 \\ \mathbf{v}_3 \cdot \mathbf{e}_1 & \mathbf{v}_3 \cdot \mathbf{e}_2 & \mathbf{v}_3 \cdot \mathbf{e}_3 \end{bmatrix} . \tag{S13}$$

Note that this operation is performed within the user-material subroutine at each integration point of the finite elements and at each frame step of the analysis. We emphasize that other methods proposed in the literature, where the homogenized first Piola–Kirchhoff stress tensor is computed from the total reaction force[6] in combination with periodic boundary conditions, are fundamentally wrong.

Algorithm S2 summarizes the entire computation of the homogenized stress.

Algorithm S2 Computation of the homogenized first Piola-Kirchhoff stress tensor

Require: Abaqus FEA field outputs from `<analysis>`.odb, v_f

▷ *Initializing*

$V_\mathrm{f} = 0$

$V_\mathrm{m} = 0$

▷ *Cycling on step frames*

for $n \in [1, N_\mathrm{iter}]$ **do**

  $\tilde{\mathbf{P}}_\mathrm{f} = \mathbf{0}, \mathbf{P}^*_\mathrm{f} = \mathbf{0}$

  $\mathbf{P}_\mathrm{m} = \mathbf{0}, \mathbf{P}^*_\mathrm{m} = \mathbf{0}$

▷ *Cycling on fibers*

  for $i \leftarrow 1$ to N_f **do**

    $V_\mathrm{f}^{(i)} = 0$

    $\mathbf{P}^{*(i)}_\mathrm{f} = \mathbf{0}$

▷ *Cycling on elements of each fiber*

    for $j \leftarrow 1$ to $N_\mathrm{ele,f}^{(i)}$ **do**

      Calculate local coordinate system $\{\mathbf{v}_k^{(j)}\}_{k=1,2,3}$

      Compute transformation matrix $[\mathbf{T}^{(j)}]$ ▷ See Eq. (S13)

      Compute stress tensor $\tilde{\mathbf{P}}_\mathrm{f}^{(j)}$ in global coordinate system ▷ See Eq. (S12)

      $\mathbf{P}^{*(i)}_\mathrm{f} = \mathbf{P}^{*(i)}_\mathrm{f} + V_\mathrm{f}^{(j)} \tilde{\mathbf{P}}_\mathrm{f}^{(j)}$

      $V_\mathrm{f}^{(i)} = V_\mathrm{f}^{(i)} + V_\mathrm{f}^{(j)}$

    end for

    $\mathbf{P}^*_\mathrm{f} = \mathbf{P}^*_\mathrm{f} + \mathbf{P}^{*(i)}_\mathrm{f}$

    $V_\mathrm{f} = V_\mathrm{f} + V_\mathrm{f}^{(i)}$

  end for

▷ *Cycling on elements of ground matrix*

  for $j \leftarrow 1$ to $N_\mathrm{ele,m}$ **do**

    $\mathbf{P}^*{}_\mathrm{m} = \mathbf{P}^*{}_\mathrm{m} + V_\mathrm{m}^{(j)} \mathbf{P}_\mathrm{m}^{(j)}$

    $V_\mathrm{m} = V_\mathrm{m} + V_\mathrm{m}^{(j)}$

  end for

▷ *Computing average and homogenized stress tensors*

  $\overline{\tilde{\mathbf{P}}}_\mathrm{f} = \mathbf{P}^*_\mathrm{f} / V_\mathrm{f}$ ▷ See Eq. (S8)

  $\overline{\mathbf{P}}_\mathrm{m} = \mathbf{P}^*_\mathrm{m} / V_\mathrm{m}$ ▷ See Eq. (S8)

  $\overline{\mathbf{P}} = \overline{\mathbf{P}}_\mathrm{m} + v_\mathrm{f} \overline{\tilde{\mathbf{P}}}_\mathrm{f}$ ▷ See Eq. (3)

end for

return $\overline{\mathbf{P}}, \overline{\mathbf{P}}_\mathrm{m}, \overline{\tilde{\mathbf{P}}}_\mathrm{f}$

Inverse parameter identification

Figure S2 summarizes the inverse parameter identification algorithm.

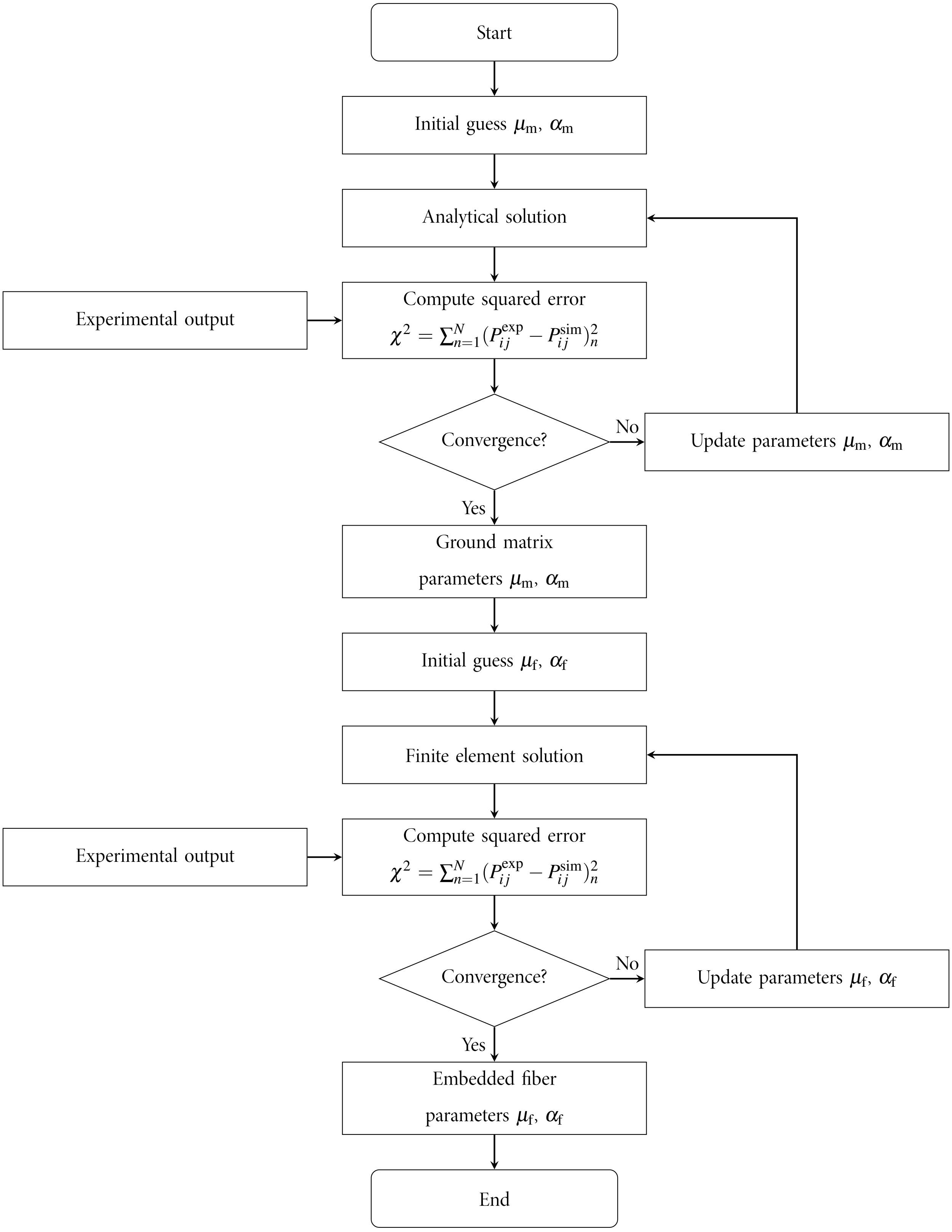

Figure S2. Flowchart of the two-step optimization process to identify the inverse parameters.

References

[1] M. Dalbosco, T. A. Carniel, E. A. Fancello, G. A. Holzapfel, Multiscale numerical analyses of arterial tissue with embedded elements in the finite strain regime, Computer Methods in Applied Mechanics and Engineering 381 (2021) 113844. doi:10.1016/j.cma.2021.113844.

[2] N. Stikov, L. M. Perry, A. Mezer, E. Rykhlevskaia, B. A. Wandell, J. M. Pauly, R. F. Dougherty, Bound pool fractions complement diffusion measures to describe white matter micro and macrostructure, NeuroImage 54 (2011) 1112–1121. doi:10.1016/j.neuroimage.2010.08.068.

[3] Dassault Systèmes SIMULIA, Abaqus 2018, Documentation, 2018.

[4] G. A. Holzapfel, Nonlinear Solid Mechanics – A Continuum Approach for Engineering, John Wiley & Sons, Chichester, UK, 2000.

[5] H. T. Garimella, R. R. Menghani, J. I. Gerber, S. Sridhar, R. H. Kraft, Embedded finite elements for modeling axonal injury, Annals of Biomedical Engineering 47 (2019) 1889–1907. doi:10.1007/s10439-018-02166-0.

[6] P. Chavoshnejad, G. K. German, M. J. Razavi, Hyperelastic material properties of axonal fibers in brain white matter, Brain Multiphysics 2 (2021) 100035. doi:10.1016/j.brain.2021.100035.
